# Pemafibrate Prevents Rupture of Angiotensin II-Induced Abdominal Aortic Aneurysms

**DOI:** 10.3389/fcvm.2022.904215

**Published:** 2022-06-30

**Authors:** Naofumi Amioka, Toru Miyoshi, Tomoko Yonezawa, Megumi Kondo, Satoshi Akagi, Masashi Yoshida, Yukihiro Saito, Kazufumi Nakamura, Hiroshi Ito

**Affiliations:** ^1^Department of Cardiovascular Medicine, Faculty of Medicine, Dentistry and Pharmaceutical Sciences, Okayama University, Okayama, Japan; ^2^Department of Molecular Biology and Biochemistry, Faculty of Medicine, Dentistry and Pharmaceutical Sciences, Okayama University, Okayama, Japan

**Keywords:** pemafibrate, angiotensin II, abdominal aortic aneurysm, oxidative stress, catalase

## Abstract

**Background:**

Abdominal aortic aneurysm (AAA) is a life-threatening disease that lacks effective preventive therapies. This study aimed to evaluate the effect of pemafibrate, a selective peroxisome proliferator-activated receptor alpha (PPARα) agonist, on AAA formation and rupture.

**Methods:**

Experimental AAA was induced by subcutaneous angiotensin II (AngII) infusion in *ApoE*^–^*^/^*^–^ mice for 4 weeks. Pemafibrate (0.1 mg/kg/day) was administered orally. Dihydroethidium staining was used to evaluate the reactive oxygen species (ROS).

**Results:**

The size of the AngII-induced AAA did not differ between pemafibrate- and vehicle-treated groups. However, a decreased mortality rate due to AAA rupture was observed in pemafibrate-treated mice. Pemafibrate ameliorated AngII-induced ROS and reduced the mRNA expression of interleukin-6 and tumor necrosis factor-α in the aortic wall. Gelatin zymography analysis demonstrated significant inhibition of matrix metalloproteinase-2 activity by pemafibrate. AngII-induced ROS production in human vascular smooth muscle cells was inhibited by pre-treatment with pemafibrate and was accompanied by an increase in catalase activity. Small interfering RNA-mediated knockdown of catalase or PPARα significantly attenuated the anti-oxidative effect of pemafibrate.

**Conclusion:**

Pemafibrate prevented AAA rupture in a murine model, concomitant with reduced ROS, inflammation, and extracellular matrix degradation in the aortic wall. The protective effect against AAA rupture was partly mediated by the anti-oxidative effect of catalase induced by pemafibrate in the smooth muscle cells.

## Introduction

Abdominal aortic aneurysm (AAA) is characterized by progressive dilation of the abdominal aorta and associated risk of rupture and sudden death. Multiple factors are associated with the development and fatal rupture of AAA, including infiltration of macrophages that release pro-inflammatory cytokines (1), generation of reactive oxygen species (ROS) (2–5), impairment and apoptosis of vascular smooth muscle cells (VSMCs) (6), and degradation of the extracellular matrix by activated matrix metalloproteinases (MMPs) (7). Unfortunately, effective preventive treatments have not yet been established despite extensive basic and clinical research on AAA. Previous clinical studies have shown the potential of beta-blockers and angiotensin-converting enzyme inhibitors to prevent the development or rupture of AAA; however, their clinical effectiveness for AAA remains controversial (8–11).

Peroxisome proliferator-activated receptors (PPARs) are transcription factors that belong to the nuclear receptor superfamily, which includes the three following subtypes: PPARα, PPARβ/δ, and PPARγ. PPARs bind to PPAR-responsive regulatory elements and regulate energy homeostasis, insulin sensitivity, and lipid metabolism by promoting the expression of various genes (12–15). In addition, the activation of PPARα can regulate the expression of genes involved in inflammation and oxidative stress (16–18). PPARα is expressed in various types of cells in the body, including vascular component cells such as macrophages, vascular smooth muscle cells, and endothelial cells (17, 19–23). Previous studies showed that fibrates, which are PPARα agonists, decrease the production of inflammatory cytokines, infiltration of monocytes, and expression of MMP genes in the aortic wall (24–27). Fibrates have also been reported to attenuate the reduction of anti-oxidative enzymes, including superoxide dismutase and catalase, in the aortic wall impaired by diabetic stress (28). Accumulating evidence showing the counteraction of fibrates on the pathogenesis of AAA suggests the potential of PPARα as a therapeutic target for the development and rupture of AAA (29).

However, the side effects of conventional PPARα agonists, especially off-target effects such as liver damage and elevated serum creatinine levels, are major concerns in clinical practice (30–32). Recently, pemafibrate, a selective PPARα modulator, has been discovered (33, 34). Pemafibrate is more potent in activating PPARα than conventional PPARα agonists, as indicated by its lower effective concentration and higher selectivity toward PPAR subtypes with reduced off-target side effects (35, 36).

In primary studies, angiotensin II (AngII)-infused hypercholesterolemic mice are widely used to develop experimental AAA. AngII infusion has been reported to promote AAA formation in mice by inducing ROS, inflammation, and activating MMPs in the aortic wall (37–40). Thus, AngII infusion in mice is a technically facile animal model that recapitulates multiple facets of AAA in human.

In this study, we investigated the protective effect of pemafibrate on AAA formation and rupture in an experimental murine model, with a focus on AngII-induced ROS production and inflammation.

## Materials and Methods

### Animals and Treatments

**FIGURE 1:**
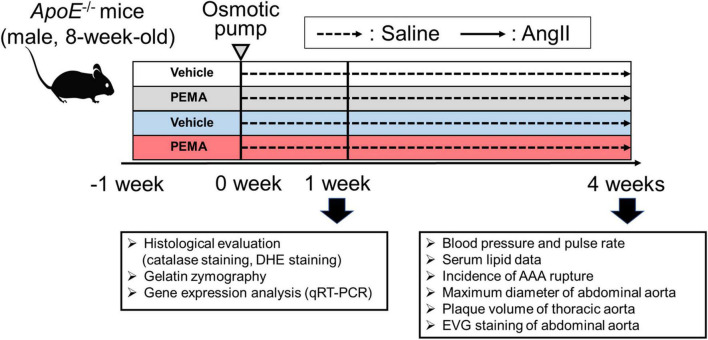
Animal experiment protocol. To develop an experimental abdominal aortic aneurysm (AAA) model, 8-week-old *ApoE-/-* male mice were infused with angiotensin II (AngII) (1,000 ng/min/kg) or saline for 4 weeks. To assess the effect of pemafibrate, treatment with vehicle or pemafibrate (PEMA) (0.1 mg/kg/day) was initiated 1 week before administration of AngII or saline, resulting in four treatment groups: vehicle-treated saline-infused (*n* = 10), pemafibrate-treated saline-infused (*n* = 10), vehicle-treated AngII-infused (*n* = 25), and pemafibrate-treated AngII-infused (*n* = 25). Five mice in each group were euthanized to evaluate the early changes after AngII infusion. Blood pressure and heart rate were measured using the tail-cuff method before and 4 weeks after AngII infusion. All mice were euthanized at their respective endpoints under anesthesia. DHE, dihydroethidium; EVG, Elastica van Gieson; qRT-PCR, quantitative reverse transcription polymerase chain reaction.

All animal experiments were conducted in accordance with experimental protocols approved by the Institutional Animal Care and Use Committee of Okayama University (OKU-2021372). Male *ApoE^–/–^* mice were purchased from Jackson Laboratory (Bar Harbor, ME, United States). Figure 1 shows the experimental animal protocol. For the AAA model, 8-week-old *ApoE^–/–^* male mice were stimulated with a continuous infusion of AngII (1,000 ng/min/kg) for 4 weeks. AngII was dissolved in sterile saline and infused using Alzet osmotic pumps (Model 2004, Durect Corp., Cupertino, United States). Osmotic pumps filled with AngII were implanted subcutaneously in the neck under ketamine and xylazine anesthesia. A saline infusion was used as the control. To evaluate the effect of pemafibrate on AAA, treatment with pemafibrate (0.1 mg/kg/day) or vehicle was commenced a week before the administration of AngII or saline. Mice were anesthetized by intraperitoneal injection of ketamine (80 mg/kg) and xylazine (5 mg/kg) before euthanization. To evaluate early changes in the aortic wall after AngII infusion, another set of mice was euthanized at 1 week. Catalase staining, dihydroethidium (DHE) staining, gelatin zymography, and gene expression analysis were performed in mice euthanized at 1 week. In addition, the evaluation of blood pressure, serum lipid profile, the incidence of AAA rupture, the maximum diameter of the abdominal aortas, plaque volume of the thoracic aorta, and histology of the abdominal aorta [elastin van Gieson (EVG) staining] were performed in mice euthanized at 4 weeks. The sample size of each group for the 1-week infusion of saline or AngII was 5. The sample size of each treatment group infused with saline or AngII for 4 weeks was as follows: vehicle-treated saline-infused mice (*n* = 10), pemafibrate-treated saline-infused mice (*n* = 10), vehicle-treated AngII-infused mice (*n* = 25), and pemafibrate-treated AngII-infused mice (*n* = 25). All mice were euthanized at their respective endpoints under anesthesia.

### Measurement of Blood Pressure

Blood pressure was measured using the tail-cuff method (MK-2000; Muromachi, Tokyo, Japan) at the start of the experiment (baseline) and 4 weeks after AngII infusion.

### Analysis of Serum Lipid Profile

Mice were fasted overnight at the endpoint of this study (4 weeks after the beginning of AngII or saline infusion). Blood was collected from the right ventricle. Serum samples were separated by centrifugation of blood samples at 3,000 rpm for 20 min. The serum was stored at -80°C. The general serum lipid profile was analyzed using high-performance liquid chromatography (Skylight Biotech Inc., Akita, Japan).

### Evaluation of the Incidence of Aortic Rupture, Abdominal Aortic Diameter, and Extent of Atherosclerosis

To assess the incidence of aortic rupture, each mouse was monitored intensively every day to ensure immediate dissection of dead mice. After termination, the abdominal aortic diameter was measured at the suprarenal lesion of the aorta using *ex vivo* imaging. In addition, thoracic aortas were used for Oil Red O staining (Sigma-Aldrich, St. Louis, MO, United States) to compare the percentage of plaque areas among the treatment groups. The plaque area was quantified using the ImageJ software (National Institutes of Health, Bethesda, MD, United States).

### Histological Assessment

Suprarenal abdominal aortic segments were fixed in 4% paraformaldehyde, embedded in paraffin, and cut into 5-μm-thick sections. Three sets of serial sections obtained at 500 μm intervals were stained with EVG using a standard protocol to evaluate the abdominal aorta longitudinally. To detect catalase in the aortic wall, immunostaining was performed using the following method. First, deparaffinized tissue sections were incubated with 0.3% H_2_O_2_ in Tris buffer for 15 min at room temperature (RT), 0.025% Triton in Tris buffer for three times, and streptavidin/biotin blocking kit (SP-2002, Vector Labs, Burlingame, CA, United States) for 15 min, respectively, at RT. Subsequently, the slides were incubated with blocking solution (X0909, DAKO, Santa Clara, CA, United States) for 15 min at RT and then incubated with a rabbit anti-mouse catalase primary antibody (1 μg/mL, ab16731, Abcam, Cambridge, United States) overnight at 4°C. Next, the sections were incubated for 30 min at room temperature with a biotinylated swine anti-rabbit IgG secondary antibody (1:500, E0353, DAKO). Finally, a biotinylated protein detection kit (SA-5704, Vector Labs) and diaminobenzidine (DAB) substrate (SK-4105, Vector Labs) were used. EVG- and catalase-stained samples were photographed using an Axioskop 2 Plus light microscope (Zeiss, Oberkochen, Germany). The percentage of catalase-positive areas in the tunica media in five different fields and 7–8 different samples were analyzed using ImageJ software.

### Cell Culture

Human aortic VSMCs were obtained from Lonza (Basel, Germany) and cultured in SmGM-2 Bullet Kit medium (Lonza) supplemented with 5% fetal bovine serum (FBS), 0.2% human fibroblast growth factor-B, 0.1% gentamicin/amphotericin B solution, 0.1% human epidermal growth factor, and 0.1% insulin. To analyze the effect of pemafibrate on ROS, cells were first grown in a reduced-serum medium for 24 h and then cultured in the respective basal medium supplemented with 1% FBS. The cells were treated with pemafibrate (0.1–10 μM) or vehicle (dimethyl sulfoxide) for 24 h. To evaluate the anti-oxidative effect of catalase activity induced by pemafibrate, 3-amino-1,2,4-triazole (3AT) (Sigma Aldrich) (50 mM), a catalase inhibitor, was co-administered with pemafibrate.

### Small Interfering Ribonucleic Acid Transfection

Gene silencing experiments were performed by transfecting VSMCs with 10 μM small interfering ribonucleic acid (siRNAs) (Ambion, Life Technologies, Darmstadt, Germany) targeting catalase (*CAT*), *PPAR*α, or the negative control, using Lipofectamine RNAiMAX (Invitrogen, Carlsbad, CA, United States), and Opti-MEM (Gibco, Waltham, MA, United States) for 24 h, before each treatment.

### Catalase Activity

The catalase activity of VSMCs was determined using a simple visual assay as described previously (41). Briefly, catalase powder (Sigma Aldrich) dissolved in 100 μL of distilled water was used to prepare catalase standards. Next, VSMCs (1.0 × 10^7^ cells) were suspended in 100 μL of phosphate-buffered saline (PBS). Catalase standard or sample solution (100 μL) was transferred to a Pyrex test tube (13 mm diameter × 100 mm height), and 100 μL each of 1% Triton X-100 (MP Biomedicals, Santa Ana, CA, United States) and 30% hydrogen peroxide (Wako, Osaka, Japan) were added, mixed gently, and incubated at 20°C. After the completion of the reaction, the height of the O_2_-forming foam, which remained constant for 15 min, was measured in millimeters using a ruler.

### Detection of Reactive Oxygen Species

The presence of ROS in the aortic walls of mice and VSMCs was analyzed using DHE staining (Molecular Probes, Eugene, United States). Briefly, VSMCs were plated on glass coverslips placed in 12-well plates. Subsequently, sub-confluent cells were stimulated with AngII (100 nM) for 30 min, washed with PBS, incubated with DHE (5 μM) for 30 min, and analyzed for fluorescence. Red fluorescence intensity (FI) (585 nm) was measured using an OLYMPUS IX71 fluorescence microscope (Tokyo, Japan). The mean FIs of 10–20 nuclei per image, 5 images per coverslip, and 3 coverslips per sample were measured using the ImageJ software. The aortas of mice treated for 1 week were perfused with PBS (pH 7.4) for 5 min at 4°C. Subsequently, the aortic tissue was harvested from the abdominal aorta, embedded in Tissue-Tek O.C.T. Compound (Sakura Finetek United States, Torrance, CA, United States), and snap-frozen. Freshly cut frozen aortic sections (5 μm) were incubated with DHE for 30 min at 37°C to detect ROS. For *in vivo* experiments, the mean FI of 10–20 nuclei per image and five images per sample were analyzed using the ImageJ software.

### Gelatin Zymography

The enzymatic activities of MMP-2 and MMP-9 were analyzed in the aortic walls of mice treated with vehicle or pemafibrate for 1 week. Briefly, 10 μg of total protein isolated from the abdominal aorta was subjected to 10% sodium dodecyl sulfate-polyacrylamide gel electrophoresis containing 1 mg/mL gelatin added before heating. After electrophoresis, the gel was washed with 2.5% Triton X-100 solution for 30 min and then incubated in a solution containing 50 mM Tris-HCl, 5 mM CaCl_2_, and 1 μM ZnCl_2_ for 16 h at 37°C. Following incubation, the gel was stained with 0.05% Coomassie Brilliant Blue R-250 for 30 min at room temperature, washed with buffer, and photographed. Pro-MMP-2, active MMP-2, and pro-MMP-9 were visualized as colorless bands against a blue background. The color density of the band formation area was determined using the ImageJ software.

### Quantitative Reverse Transcription-Polymerase Chain Reaction

RNA was extracted from the aortic tissue of mice in the acute model or from VSMCs using the RNeasy Mini Kit (Qiagen, Valencia, United States). Complementary diribonucleic acid (cDNA) was synthesized from 1.0 μg of extracted total RNA using ReverTra Ace (TOYOBO, Osaka, Japan). The synthesized cDNAs were subjected to polymerase chain reaction (PCR) using the TaqMan Gene Expression Master Mix (Applied Biosystems, Foster City, United States) and predesigned gene-specific primer and probe sets (TaqMan Gene Expression Assays; Applied Biosystems). TaqMan gene expression probe-and-primer sets for *PPAR*α, superoxide dismutases (*SOD1* and *SOD2)*, nicotinamide adenine dinucleotide phosphate oxidases (*NOX2* and *NOX4*), *CAT*, heme oxygenase-1 (*HO-1*), interleukin-6 (*IL-6*), tumor necrosis factor-α (*TNF*α), and transforming growth factor-β1 (*TGF-*β*1*) were purchased from Applied Biosystems. Real-time PCR was performed in triplicate for each sample using the QuantStudio 1 real-time PCR System (Applied Biosystems) described previously (42). The results were quantified using the relative Ct method and normalized to the internal control, glyceraldehyde 3-phosphate dehydrogenase. See Supplementary Table 1 for PCR primer details.

### Statistical Analyses

All analyses were performed using EZR version 1.41 (Saitama Medical Center, Jichi Medical University, Saitama, Japan) (43), a graphical user interface of R (The R Foundation for Statistical Computing, Vienna, Austria), or SigmaPlot version 14.5 (Systat Software Inc., San Jose, CA, United States). For normally distributed continuous variables, results are expressed as the mean ± standard error of the mean. One-way analysis of variance with Bonferroni *post-hoc* test was used to examine the differences among groups. Non-normally distributed continuous variables were expressed as median (interquartile range) and analyzed using the Kruskal–Wallis tests. Categorical variables are presented as absolute values and frequencies, and categorical variables were compared using the chi-square test. Kaplan–Meier survival curves were used to evaluate survival rates, and significance was assessed using the log-rank test. In some experiments, technical replicates were performed as described in each figure legend. Differences with *p* < 0.05 were considered statistically significant.

## Results

### Pemafibrate Reduced Angiotensin II-Induced Abdominal Aortic Rupture

**FIGURE 2:**
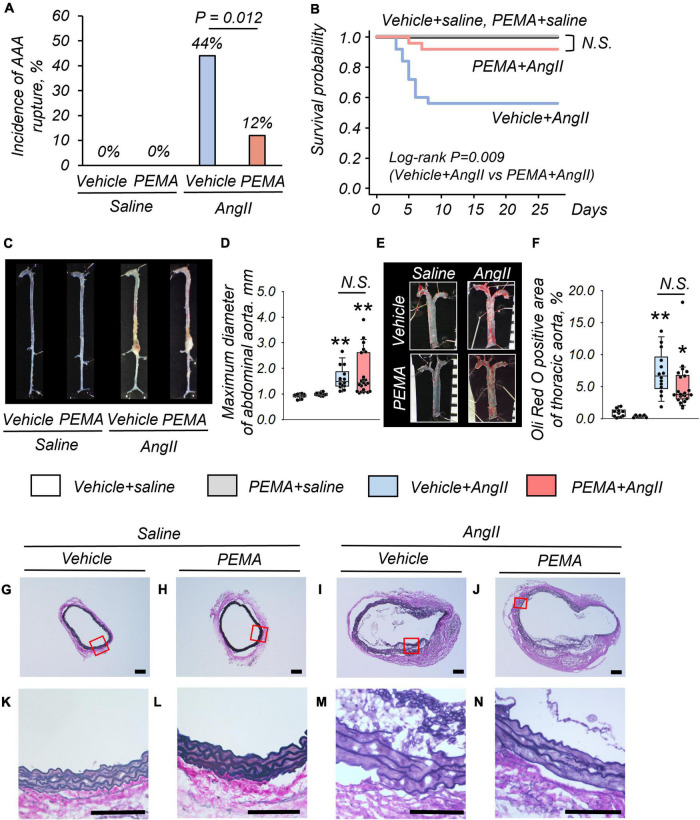
Impact of pemafibrate (PEMA) on abdominal aortic aneurysm (AAA) in a murine model. Incidence of aortic rupture in the vehicle-treated saline-infused (vehicle + saline) (*n* = 10), PEMA-treated saline-infused (PEMA + saline) (*n* = 10), vehicle-treated angiotensin II-infused (vehicle + AngII) (*n* = 25), and PEMA-treated angiotensin II-infused (PEMA + AngII) (*n* = 25) groups **(A)**. Kaplan–Meier survival curve for AngII- or saline-infused mice in the presence or absence of PEMA **(B)**. Representative findings of aortas **(C)** and maximum diameter of suprarenal abdominal aortas **(D)** extracted from surviving mice. Representative findings of Oil Red O staining **(E)** and Oil Red O-positive area **(F)** of thoracic aortas extracted from surviving mice [*n* = 10 (vehicle + saline group), *n* = 10 (PEMA + saline group), *n* = 14 (vehicle + AngII group), and *n* = 22 (PEMA + AngII group)]. Representative results of Elastica van Gibson staining **(G–N)**. Data are expressed as frequencies for categorical variables and analyzed by chi-square test **(A)** or as median (interquartile range) and analyzed using the Kruskal–Wallis test followed by Bonferroni corrections **(D,F)**. Survival probabilities were compared using the log-rank test **(B)**. *p < 0.01 vs. vehicle + saline group; **p < 0.001 vs. vehicle + saline. N.S., not significant. Scale bar = 100 μm.

At 4 weeks, no fatal aortic rupture was observed in vehicle- or pemafibrate-treated mice without AngII infusion (Figure 2A). The incidence of fatal aortic rupture in the pemafibrate-treated AngII-infused group was significantly lower than that in the vehicle-treated AngII-infused group [3/25 (12%) vs. 11/25 (44%), *p* = 0.012]. Immediate dissection of dead mice confirmed that all ruptures had occurred in the suprarenal abdominal aorta. Kaplan–Meier analysis indicated significantly increased survival in the pemafibrate-treated AngII-infused group compared with that in the vehicle-treated AngII-infused group (log-rank *p* = 0.009) (Figure 2B). The maximum abdominal aortic diameter of surviving mice was significantly increased by AngII infusion; however, this increase was not significantly changed by pemafibrate at 4 weeks (Figures 2C,D). Figure 2E shows representative findings of the thoracic aorta stained with Oil Red O. There was no significant difference in the percentage of plaque area between the vehicle- and pemafibrate-treated AngII-infused mice (Figure 2F). Representative findings of EVG staining of the abdominal aorta at 4 weeks are shown in Figures 2G–N. Degradation of the elastic lamina was observed in the aortic walls of vehicle- and pemafibrate-treated AngII-infused mice.

### Pemafibrate Did Not Influence Serum Lipid Profiles and Blood Pressure

**FIGURE 3:**
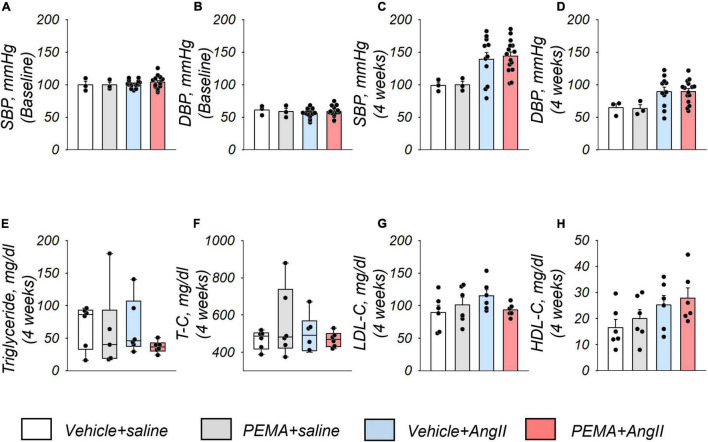
Blood pressure and serum lipid profiles of study mice. Systolic blood pressure (SBP) and diastolic blood pressure (DBP) at the start of the experiment (baseline) **(A,B)** and 4 weeks after the administration of AngII or saline infusion **(C,D)** in each group. Sample size: *n* = 3 (vehicle + saline and PEMA + saline group] and *n* = 11–16 (vehicle + AngII and PEMA + AngII group) in triplicate. Samples that matched the median body weights of each group were selected. Serum levels of triglycerides, total cholesterol (T-C), low-density lipoprotein cholesterol (LDL-C), and high-density lipoprotein cholesterol (HDL-C) 4 weeks after the administration of AngII or saline infusion in each group **(E–H)**, respectively. Sample size: *n* = 6 per group. Samples that matched the median body weights of each group were selected. Data are expressed as mean ± standard error of the mean and analyzed by one-way analysis of variance for normally distributed continuous variables **(A–D,G,H)** or expressed as median (interquartile range) and analyzed using the Kruskal–Wallis test **(E,F)**. *p* < 0.05.

Compared with the baseline of the experiment (Figures 3A,B), an increase in systolic and diastolic blood pressure by AngII infusion was observed at 4 weeks (Figures 3C,D). Pemafibrate did not ameliorate this increase in blood pressure. Analyses of serum triglyceride, total cholesterol, low-density lipoprotein cholesterol, and high-density lipoprotein cholesterol levels indicated no significant difference among all groups of mice at 4 weeks (Figures 3E–H).

### Pemafibrate Increased the Expression of Catalase and Reduced Angiotensin II-Induced Reactive Oxygen Species in the Aortic Wall

**FIGURE 4:**
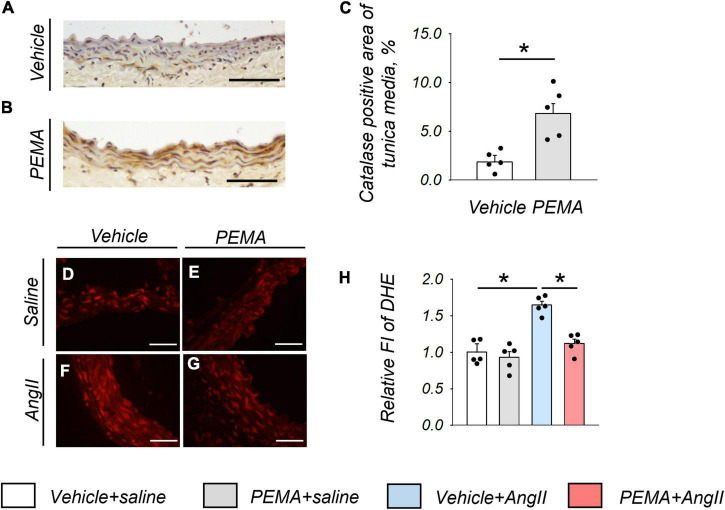
Catalase expression and the reduction in reactive oxygen species (ROS) in the aortic wall at 1 week after angiotensin (AngII) infusion. Representative findings of anti-catalase staining **(A,B)** and quantitative evaluation of catalase expression **(C)** in the suprarenal aorta from vehicle-treated saline-infused mice and in pemafibrate (PEMA)-treated saline-infused mice (*n* = 5 per group, performed in duplicate). Representative results of dihydroethidium (DHE) staining **(D–G)** and relative fluorescence intensity (FI) of DHE staining (*n* = 5 per group, performed in duplicate) **(H)** in the suprarenal aorta extracted from vehicle-treated saline-infused, PEMA-treated saline-infused, vehicle-treated AngII-infused, and PEMA-treated AngII-infused mice. Data are expressed as the mean ± standard error of the mean. All analyses were performed using analysis of variance and Bonferroni corrections. *p < 0.01. Scale bar = 50 μm.

Representative findings of anti-catalase staining of abdominal aortic tissue extracted from mice at 1 week after AngII infusion are shown in Figures 4A,B. Pemafibrate significantly increased the expression of catalase in the tunica media of the aortic wall compared with that in control mice (Figure 4C). Figures 4D–G represents DHE staining findings in the tunica media of abdominal aorta extracted from mice 1 week after AngII infusion. In addition, pemafibrate pre-treatment significantly attenuated the increase in ROS induced by AngII, as indicated by FI (Figure 4H).

### Pemafibrate Enhanced the Expression of Catalase and Suppressed the Expression of Genes Associated With Inflammation in the Aortic Tissue

**FIGURE 5:**
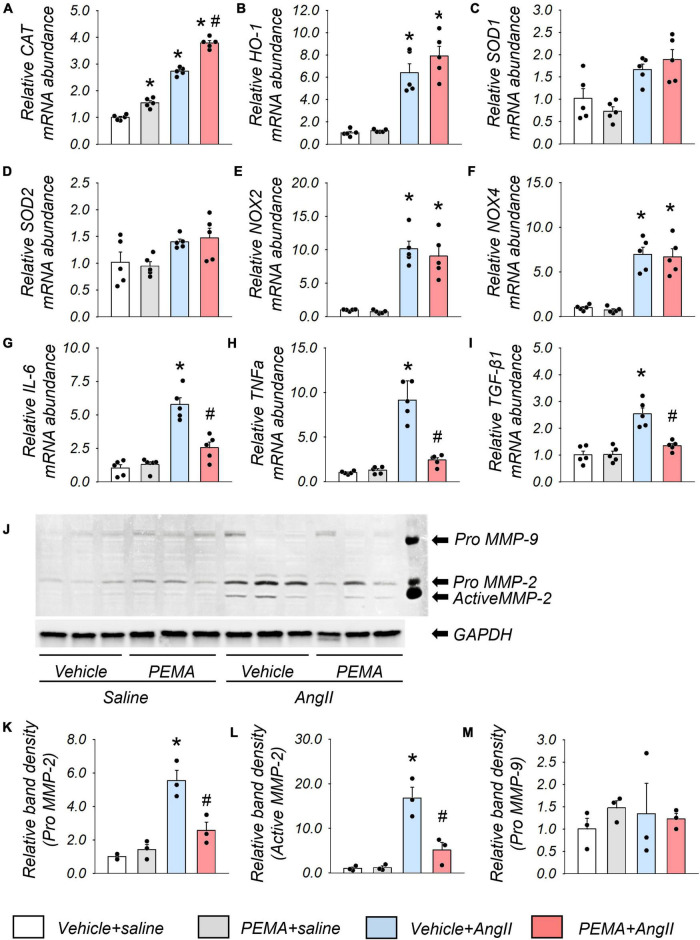
Gene expression and matrix metalloproteinase (MMP) activity in the aortic tissues extracted from the acute model of ApoE^–/–^ mice. **(A–I)** mRNA expression levels of catalase (*CAT*), heme oxygenase-1 (*HO-1)*, superoxide dismutase 1 (*SOD-1*), *SOD-2*, nicotinamide adenine dinucleotide phosphate oxidases (*NOX2* and *NOX4*), Interleukin-6 (*IL-6*), tumor necrosis factor-α (*TNF*α), and transforming growth factor-β1 (*TGF-*β*1*) in the suprarenal aortic tissues extracted from vehicle-treated saline-infused (vehicle + saline), pemafibrate-treated saline-infused (PEMA + saline), vehicle-treated angiotensin II-infused (vehicle + AngII), and PEMA-treated angiotensin II-infused (PEMA + AngII) mice (n = 5 per group, performed in duplicates). All gene expression analyses were conducted using quantitative reverse transcription-polymerase chain reaction, and each gene expression level was normalized using glyceraldehyde 3-phosphate dehydrogenase (*GAPDH*). **(J)** MMP-2, and MMP-9 activities in the abdominal aorta of mice infused with saline or AngII for 4 weeks were analyzed using gelatin zymography. Quantitative analysis of pro-MMP-2 **(K)**, active MMP-2 **(L)**, and pro-MMP-9 **(M)** in the abdominal aorta (*n* = 3 per group, performed in duplicates). For gelatin zymography, the samples matched to the median body weight of each group were selected. Data are expressed as mean ± standard error of the mean. All analyses were performed using analysis of variance and Bonferroni corrections. *p < 0.05 vs. the vehicle-treated saline-infused group; ^#^*p* < 0.05 vs. the vehicle-treated AngII-infused group.

The administration of pemafibrate increased *CAT* mRNA abundance in the aortic wall of mice infused with saline for 1 week. This increase was further enhanced in mice infused with AngII (Figure 5A). The mRNA expression levels of *HO-1*, *NOX-2*, and *NOX-4* in the vehicle-treated AngII-infused group were also significantly increased compared with those in the vehicle-treated group; however, these increases were not affected by pemafibrate treatment (Figures 5B,E,F). There were no statistically significant differences in the mRNA expression level of *SOD-1* and *SOD-2* between any of the groups (Figures 5C,D). Pemafibrate significantly suppressed the enhanced mRNA expression of *IL-6, TNF-*α, and *TGF-*β*1* induced by AngII infusion (Figures 5G–I). MMP-2 activity was significantly increased by AngII infusion, and this increase was attenuated by pemafibrate treatment (Figures 5J–L). The activity of pro-MMP-9 was not affected by AngII infusion or pemafibrate treatment (Figures 5J,M).

### Pemafibrate Decreased Reactive Oxygen Species by Increasing the Expression and Activity of Catalase in Vascular Smooth Muscle Cells

**FIGURE 6:**
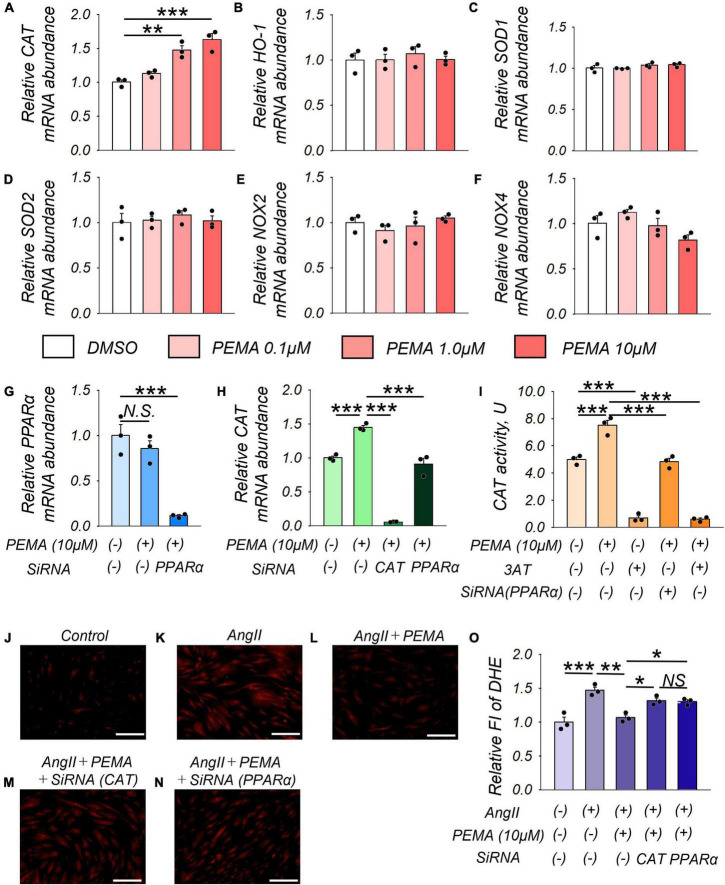
Pemafibrate (PEMA)-mediated gene expression changes and the reduction in ROS in vascular smooth muscle cells (VSMCs). **(A–F)** mRNA expression levels of catalase (*CAT*), heme oxygenase-1 (*HO-1*), superoxide dismutase 1 (*SOD-1*), *SOD-2*, nicotinamide adenine dinucleotide phosphate oxidases (*NOX2* and *NOX4*) in VSMCs treated with dimethyl sulfoxide (DMSO) and PEMA (0.1, 1.0, and 10 μM) for 24 h. **(G)** mRNA expression levels of peroxisome proliferator-activated receptor alpha (*PPAR*α) in VSMCs treated with 10 μM PEMA with or without transfection using SiRNA of *PPAR*α for 24 h before the PEMA administration. **(H)** mRNA expression levels of *PPAR*α in VSMCs treated with 10 μM PEMA with or without transfection using SiRNA of *CAT* or *PPAR*α. All gene expression analyses were conducted using quantitative reverse transcription-polymerase chain reaction, and each gene expression level was normalized using glyceraldehyde 3-phosphate dehydrogenase (*GAPDH*). **(I)** CAT activity in VSMCs (1.0 × 10^7^ cells) treated with 10 μM PEMA in the presence or absence of 3-amino-1,2,4-triazole (3AT) treatment and/or transfection using SiRNA of PPARα. Representative results of DHE staining **(J–N)** and relative DHE fluoro-intensity (FI) **(O)** of VSMCs stimulated with AngII (100 nM) with or without PEMA, and/or transfection using SiRNA of CAT or PPARα. All analyses were performed using analysis of variance and Bonferroni corrections; *n* = 3 per group, performed in duplicates. Data are expressed as mean ± standard error of the mean. *p < 0.05; **p < 0.01; ***p < 0.001. N.S., not significant.

Treatment of VSMCs with pemafibrate (10 μM) significantly enhanced *CAT* mRNA expression by 1.6-fold compared with that of the untreated control. Furthermore, this increase in expression was dose-dependent (Figure 6A). However, pemafibrate treatment did not affect the mRNA expression of any of the other genes analyzed in this study (Figures 6B–F). Pemafibrate treatment did not directly influence *PPAR*α mRNA expression, but transfection with siRNA-*PPAR*α significantly suppressed *PPAR*α mRNA expression (Figure 6G). Accordingly, the pemafibrate treatment-mediated increase in catalase expression was significantly attenuated by transfection with siRNA-*CAT* and siRNA-*PPAR*α in VSMCs (Figure 6H). Catalase activity was enhanced in pemafibrate-treated VSMCs compared with that in the untreated control. This increase in catalase activity was suppressed by adding 3AT, a catalase inhibitor, and was attenuated by siRNA-*PPAR*α (Figure 6I). DHE staining of VSMCs treated with a combination of AngII, pemafibrate, and siRNA of *CAT* or *PPAR*α is shown in Figures 1J–N. Stimulation with AngII significantly increased ROS by 1.5-fold, which was attenuated by pemafibrate treatment. This effect of pemafibrate treatment was reversed to a moderate extent by transfection with siRNA-*CAT* or siRNA-*PPAR*α in VSMCs (Figure 6O).

## Discussion

To the best of our knowledge, this is the first study to demonstrate the protective effect of pemafibrate on the prevention of fatal aortic rupture in an experimental AAA model. The primary finding of this study was that pemafibrate did not ameliorate the size of AngII-induced AAAs but significantly prevented fatal aortic rupture, which may be mediated by its anti-oxidative and anti-inflammatory effects. This protective effect against aortic rupture is partly attributable to the enhanced expression and activity of catalase in VSMCs.

Catalase, a strong antioxidant enzyme, mitigates oxidative stress by converting cellular hydrogen peroxide to water and oxygen. Overexpression of the catalase gene is associated with decreased inflammatory markers, VSMC apoptosis, and MMP activity in the aorta, leading to the prevention of experimental AAA formation in mice (44). PPARα agonists increase the expression and activity of catalase in diverse tissues, including the heart, liver, and kidneys (16, 45–47). In the present study, we demonstrated that pemafibrate treatment significantly decreased ROS levels in AngII-stimulated aortic tissue of mice and VSMCs, both of which were associated with increased catalase gene expression. The ability of pemafibrate to increase catalase activity and gene expression, leading to decreased ROS, was attenuated by knocking down *PPAR*α in VSMCs. PPARα-associated enhanced expression of the catalase gene by pemafibrate is further supported by the presence of PPAR-α-specific binding sites in the promoter region of catalase (48). Pemafibrate-induced reduction in ROS could have contributed to the reduced incidence of aortic rupture in experimental AAA. However, ROS include several components such as superoxide anions and hydrogen peroxide. As DHE staining is mainly used to detect superoxide, further studies are needed to elucidate the impact of other ROS on aortic rupture in experimental AAA.

Inflammation plays a pivotal role in developing cardiovascular diseases, including AAA (49, 50). A previous study showed that pro-inflammatory cytokines, including IL-6, IL-β1, TNF-α, monocyte chemoattractant protein (MCP)-1, and MCP-2, may contribute to pathological changes within the established, pre-ruptured AAA (51). During AAA development, monocytes are recruited into the aortic wall by chemotactic cytokines, including IL-6 and MCP-1 (52). Macrophages produce MMPs, cytokines, and chemokines in mouse and human AAA lesions (1). MMPs secreted by inflammatory cells induce ECM degradation, resulting in a decrease in aortic wall integrity. Our study demonstrated that pemafibrate significantly suppressed the enhanced gene expression of the pro-inflammatory cytokines IL-6, TNF-α, and TGF-β1 in the aortic wall 1 week after AngII infusion. Several studies have reported the preventive effects of PPARα on inflammation and atherosclerosis (53–56). One study showed that PPARα activation could decrease the production of IL-6 (54) and the cytokine-induced expression of adhesion molecules, such as vascular cell adhesion molecule-1, both *in vivo* and *ex vivo* (55). However, few studies have demonstrated the anti-inflammatory effects of pemafibrate on the aortic wall. Administration of pemafibrate decreased the gene expression of vascular cell adhesion molecule-1 and IL-6 in atherosclerotic lesions of *ApoE2*-knock-in mice (53). Previous studies have suggested that pemafibrate may affect the polarization or migration of macrophages through PPARα activation-mediated regulation of gene expression.

Furthermore, pemafibrate attenuated AngII-induced enhancement of MMP-2 activity in the aortic wall of mice. Maintenance of the structural integrity of the aortic wall, together with inhibition of extracellular matrix degradation, is primarily responsible for preventing fatal aortic rupture in AAA (57). ROS are key modulators of MMP activity (37). Accordingly, suppression of ROS in VSMCs protects against AAA formation in mice (58). In this study, MMP-2 activity after 1 week of treatment was significantly reduced in pemafibrate-treated mice compared with that in vehicle-treated mice. Therefore, it is speculated that pemafibrate might prevent extracellular matrix degradation at the early stages in the murine AAA model, thereby ameliorating fatal abdominal aortic rupture.

Pemafibrate is widely used to treat hypertriglyceridemia owing to its high clinical efficacy and minor disadvantages compared with those of conventional PPARα agonists (59). Furthermore, PROMINENT, a large ongoing randomized controlled trial, is investigating the preventive effect of pemafibrate on cardiovascular events in patients with type 2 diabetes mellitus (60). Considering that pemafibrate improved survival in AngII-infused mice by preventing fatal rupture of AAA, it may provide a promising novel treatment strategy for AAA in clinical practice.

This study had several limitations. First, the effects of pemafibrate on different experimental AAA models were not evaluated. It is well known that the AngII-induced AAA model is associated with a higher rate of aortic rupture than other experimental models of AAA induced by CaCl_2_, CaPO_4_, or elastase (61). Although it is possible that pemafibrate can attenuate the dilatation and rupture of the abdominal aorta in other AAA models, further studies are required to confirm the reduction in the rupture rate in animal models. Second, this study examined the protection, not regression, of AAA caused by pemafibrate. In clinical practice, treatment is initiated after the establishment of AAA. Prospective clinical studies are required to determine the beneficial effects of pemafibrate on AAA. Third, we did not directly compare the effects of conventional PPARα agonists and pemafibrate on the development and aortic rupture of AAA. It has been reported that fenofibrate, a major conventional PPARα agonist, reduces aortic dilatation in murine models of aortic aneurysms (62, 63). The difference in the effects of pemafibrate and other PPARα agonists on aortic rupture needs to be assessed in the same AAA model. Fourth, we did not examine changes in the lipid profile during treatment. According to previous reports, AngII can affect the plasma levels of triglycerides in rodent models (64, 65). Furthermore, another study showed that serum triglyceride levels were a risk factor for AAA rupture (66). However, in this study, as there were no significant differences in serum lipid profile among the study groups at 4 weeks, serum triglyceride levels may not significantly affect aortic rupture.

## Conclusion

Pemafibrate significantly prevented aortic rupture in a murine AAA model, concomitant with decreased ROS levels and gene expression of pro-inflammatory cytokines. In addition, pemafibrate-mediated increase in catalase gene expression and activity might be a novel contributing factor associated with its beneficial effects in AAA. Our results suggest that pemafibrate-mediated PPARα modulation is a promising drug for the prevention of AAA rupture.

## Data Availability Statement

The raw data supporting the conclusions of this article will be made available by the authors, without undue reservation.

## Ethics Statement

The animal study was reviewed and approved by the Institutional Animal Care and Use Committee, Okayama University.

## Author Contributions

TM conceptualized the study and acquired funding. NA, TM, TY, MY, MK, YS, and KN designed the methodology. NA, TY, and MK conducted experiments under the supervision of HI. NA wrote the first draft of this manuscript. TM, TY, MY, YS, KN, and HI reviewed and edited the manuscript. All authors contributed to the article and approved the submitted version.

## Conflict of Interest

The authors declare that the research was conducted in the absence of any commercial or financial relationships that could be construed as a potential conflict of interest.

## Publisher’s Note

All claims expressed in this article are solely those of the authors and do not necessarily represent those of their affiliated organizations, or those of the publisher, the editors and the reviewers. Any product that may be evaluated in this article, or claim that may be made by its manufacturer, is not guaranteed or endorsed by the publisher.
