# Supplementary material for: Pemafibrate Prevents Rupture of Angiotensin II-Induced Abdominal Aortic Aneurysms
Source: Front Cardiovasc Med. 2022 Jun 30;9:904215. doi: 10.3389/fcvm.2022.904215 (PMC9280056; doi:10.3389/fcvm.2022.904215)
Supplement: Supplementary file 1 [file Table_1.DOCX]

**SUPPLEMENTAL MATERIAL**

**Pemafibrate prevents rupture of angiotensin II-induced abdominal aortic aneurysms**

Naofumi Amioka^a^, Toru Miyoshi^a^, Tomoko Yonezawa^b^, Megumi Kondo^a^, Satoshi Akagi^a^, Masashi Yoshida^a^, Yukihiro Saito^a^, Kazufumi Nakamura^a^, Hiroshi Ito^a^

^a^ Department of Cardiovascular Medicine, Okayama University Graduate School of Medicine, Dentistry and Pharmaceutical Science, Okayama, Japan

^b^ Department of Molecular Biology and Biochemistry, Okayama University Graduate School of Medicine, Dentistry and Pharmaceutical Science, Okayama, Japan

**Address for correspondence:**

Toru Miyoshi, MD, PhD

Department of Cardiovascular Medicine, Okayama University Graduate School of Medicine, Dentistry and Pharmaceutical Sciences, 2-5-1 Shikata-cho, Kita-ku, Okayama 700-8558, Japan

Phone: +81-86-235-7351; FAX: +81-86-235-7353; E-mail: miyoshit@cc.okayama-u.ac.jp

**Keywords:** pemafibrate, angiotensin II, abdominal aortic aneurysm, oxidative stress, catalase

**Supplemental Table I: Taqman Assay Probes**

| **Gene** | **Species** | **Vendor or Source** | **Assay ID** |
| --- | --- | --- | --- |
| *PPARα* | Human | Applied Biosystems | Hs00947536_m1 |
| *SOD1* | Human | Applied Biosystems | Hs00533490_m1 |
|  | Mouse | Applied Biosystems | Hs00533490_m1 |
| *SOD2* | Human | Applied Biosystems | Hs00167309_m1 |
|  | Mouse | Applied Biosystems | Mm01313000_m1 |
| *NOX2* | Human | Applied Biosystems | Hs00166163_m1 |
|  | Mouse | Applied Biosystems | Mm01287743_m1 |
| *NOX4* | Human | Applied Biosystems | Hs04980925_m1 |
|  | Mouse | Applied Biosystems | Mm00479246_m1 |
| *CAT* | Human | Applied Biosystems | Hs00156308_m1 |
|  | Mouse | Applied Biosystems | Mm00437992_m1 |
| *HO-1* | Human | Applied Biosystems | Hs01110250_m1 |
|  | Mouse | Applied Biosystems | Mm00516005_m1 |
| *IL-6* | Mouse | Applied Biosystems | Mm00446190_m1 |
| *TNFα* | Mouse | Applied Biosystems | Mm00443258_m1 |
| *TGF-β1* | Mouse | Applied Biosystems | Mm01178820_m1 |
| *GAPDH* | Human | Applied Biosystems | Hs02786624_g1 |
|  | Mouse | Applied Biosystems | Mm99999915_g1 |
